# Reliability of Bigliani’s Classification using Magnetic Resonance Imaging for Determination of Acromial Morphology

**DOI:** 10.5704/MOJ.2211.008

**Published:** 2022-11

**Authors:** K Sahin, AS Kendirci, E Kocazeybek, N Demir, Y Saglam, A Ersen

**Affiliations:** 1 Department of Orthopedics and Traumatology, Mus State Hospital, Mus, Turkiye; 2 Department of Orthopedics and Traumatology, Istanbul University Istanbul, Istanbul, Turkiye; 3 Department of Orthopedics and Traumatology, Biruni University, Istanbul, Turkiye

**Keywords:** Bigliani’s classification, acromion, subacromial impingement syndrome, acromial morphology, magnetic resonance imaging

## Abstract

**Introduction:**

Bigliani classification is used for determination of acromial morphology, but poor inter-observer reliability has been reported on conventional radiographs. This study aims to assess inter- and intra-observer reliability using magnetic resonance imaging (MRI).

**Materials and methods:**

Forty consecutive patients diagnosed with subacromial impingement syndrome were included to study. All subjects underwent standard shoulder MRI scan and acromial shape was evaluated by nine observers of different level of expertise (three attending surgeons, three senior orthopaedic residents and three radiologists). A second set of evaluation was performed in order to assess intra-observer reproducibility. Kappa (κ) coefficient analyses both for interobserver reliability and intra-observer reproducibility were then performed.

**Results:**

Overall inter-observer agreement among nine observers was fair (κ=0.323). κ values for all 4 individual types ranged from 0.234 to 0.720 with highest agreement for type 4 and lowest agreement for type 3. Second evaluation did not result with an increase of inter-observer agreement (κ=0.338, fair). The κ coefficients for intra-observer reproducibility of nine observers ranged from 0.496 to 0.867. Overall intra-observer reproducibility was substantial. Comparison of inter- and intra-observer reliability among three groups showed no significant difference (p=0.92 and 0.22, respectively).

**Conclusion:**

Results showed that MRI did not show superior reliability compared to conventional radiographs. Moreover, inter- and intra-observer agreement did not differ between observers of different level of expertise. Findings of present study suggest that despite a sophisticated imaging modality like MRI, Bigliani’s classification apparently lacks accuracy and additional criteria, or different assessment methods are required to assess acromial morphology for clinical guidance.

## Introduction

**Fig. 1.**
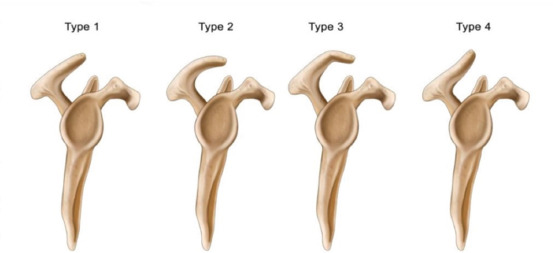
Illustration showing modified Bigliani’s classification.

Rotator cuff disorders depict a wide spectrum of pathologic conditions ranging from subacromial impingement syndrome (SIS) to cuff tear arthropathy. The aetiology is multifactorial and there are various theories regarding the pathogenesis of rotator cuff disorders. The factors leading to these conditions can be categorised in intrinsic and extrinsic factors^1-4^. Even though the relationship of causality is controversial between acromion morphology and rotator cuff disorders, abutment of coracoacromial arch against the underlying rotator cuff tendons was suggested as a major extrinsic factor causing SIS and rotator cuff tear^5^. Therefore, a considerable emphasis has been placed on understanding the anatomy of coracoacromial arch and acromion morphology. In this context, the first and most widely used classification system was reported by Bigliani *et al*^6^ who also described the clinical significance of acromial shape. The morphologic condition was classified as type I (flat), type II (curved) or type III (hooked) by evaluating the acromial shape on outlet views. Subsequently, a modification of this classification was proposed, and a fourth type of acromial morphology (convex) was also described^7^ (Fig. 1).

Even though this classification system is widely adopted and used in clinical practice, there are significant disagreements in the literature, especially regarding the reported incidences of morphological types of acromion^6,8-10^ and that was associated to the lack of standardised objective criteria for the evaluation of acromial morphology^10^. Previous studies assessing the reliability of this classification system depicted poor inter-observer reliability which revealed a necessity of more definitive criteria to distinguish different acromion shapes and classify the morphology^9,11^. Moreover, lack of standardisation of radiographs and differences due to minor changes of patient and central beam positioning were also suggested as possible factors for poor reliability^12^. Therefore, magnetic resonance imaging (MRI) was proposed as an alternative imaging modality in order to avoid these projection errors related to conventinal radiography^12,13^. Even though the appearance of acromion morphology is highly dependent on the plane of the image, we believe that MRI based evaluation of acromial shape is more reliable with standard selection of slice position and strict assessment protocol.

The aim of this study was to evaluate reliability of Bigliani’s classification using MRI as the imaging modality and to expose the precise accuracy of this commonly used system by avoiding the disadvantages related to conventional radiographs.

## Materials and Methods

Institutional review board approval was obtained before the formation of this study. Forty consecutive patients (40 shoulders) who were diagnosed with SIS in a single university hospital between February 2017 and January 2018 with adequate MRI scans were prospectively evaluated. The patients included 17 males and 23 females and average age was 49.1 (±14.7) years. Twenty-one shoulders were right, and 19 shoulders were left. Exclusion criteria included history of previous surgery or trauma of the affected shoulder, presence of significant acromiohumeral joint or glenohumeral joint arthritis seen on radiographs or MRI, presence of any rotator cuff tear on MRI and history of documented rheumatological disease. All patients were evaluated for SIS based on clinical examination including Neer’s, Hawkins’s and Yocum’s impingement tests and with MRI findings of subacromial space narrowing, bursitis or rotator cuff tendinitis.

**Fig. 2.**
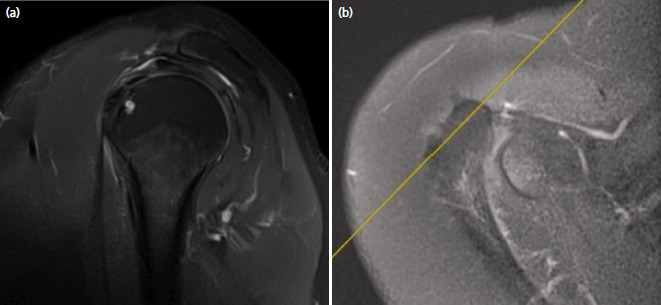
(a) Parasagittal magnetic resonance image on T2-weighted fat-suppressed sequences on which observers made evaluations. (b) Selection of adequate slice position on an axial localising image.

MRI scans were obtained with standard positioning of the arm and shoulder following the protocol determined by the institutional radiology department, using a 1.5T MR scanner equipped with a shoulder surface coil with slice thickness of 4mm and a maximum gradient capacity of 33mT/m [Siemens® Magnetom Aera, Erlangen, Germany]. Parasagittal MR images of T2-weighted fat-suppressed sequences, perpendicular to the supraspinatus tendon as determined with an axial localising image were used (Fig. 2). The slice position that was located just lateral to acromioclavicular joint was chosen from obtained MR images which was reported to be the most adequate slice position for acromial morphology assessment^12,14^.

Three attending surgeons, three senior orthopaedics residents and three attending musculoskeletal radiologists independently evaluated each subject. All observers were blinded to evaluations of other observers and any patient information. All observers were familiar with Bigliani classification, and they reviewed same schematic representation of the classification before evaluating subjects (Fig. 1). Observers were instructed to classify acromial morphology on the selected MR images of slice positions described above.

Inter-observer reliability was assessed using the comparison of the classification between the observers. In order to determine the intra-observer reproducibility, a second set of evaluation of each subject was made by all observers with at least eight weeks of separation. The patient order was rearranged between two sets of evaluation in order to avoid recall bias. Kappa (κ) coefficient analyses both for inter-observer reliability and intra-observer reproducibility were then performed. κ adjusts the amount of agreement among observers that could have occurred by chance. κ values range between +1.0 (indicating complete agreement) and -1.0 (indicating lower agreement than expected by chance). The level of agreement was then classified as described by Landis and Koch^15^ (Table I).

**Table I:** Categorisation of agreement as originally described by Landis and Koch^15^

Agreement	κ value
Slight	0.00-0.20
Fair	0.21-0.40
Moderate	0.41-0.60
Substantial	0.61-0.80
Excellent	0.81-1.00

The significance of inter- and intra-observer agreement between different groups were assessed using one-way ANOVA test. The level of significance was set at p=0.05. All statistical analyses were performed using IBM SPSS Statistics for Windows, Version 26.0 [IBM Corp. Armonk, NY].

## Results

In each evaluation of two sets, total agreement was present between all nine observers 20% of the time. Eight of nine observers agreed 7.5% of the time. The analysis revealed that overall inter-observer agreement among nine observers was fair (κ=0.323). κ values for all 4 individual types ranged from 0.234 to 0.720 with highest agreement for type 4 and lowest agreement for type 3 (Table II). Second evaluation did not result with an increase of inter-observer agreement (κ=0.338, fair). Comparison of inter-observer agreement results between three groups (attendings, residents, and radiologists) showed no significant difference (mean κ values: 0.308 vs 0.321 vs 0.319, p=0.92)

**Table II:** Average κ values and agreement for each acromion types for all nine observers

Acromion shape	κ value	Agreement
Type I	0.292	Fair
Type II	0.286	Fair
Type III	0.234	Fair
Type IV	0.720	Substantial
Overall	0.323	Fair

The κ coefficients for intra-observer reproducibility of nine observers ranged from 0.496 to 0.867 (Table III). Overall intra-observer reproducibility was substantial (κ=0.650). Results revealed that while residents had moderate intra-observer agreement (mean κ=0.555), attendings and radiologists showed substantial agreement (mean κ= 0.660 and 0.734, respectively). Comparison of intra-observer results between three groups showed no significant difference (p=0.22)

**Table III:** Intra-observer reliability of all nine observers based on two separate observations

Observer	κ value
Resident 1	0.515
Resident 2	0.575
Resident 3	0.576
Attending surgeon 1	0.496
Attending surgeon 2	0.713
Attending surgeon 3	0.770
Radiologist 1	0.867
Radiologist 2	0.623
Radiologist 3	0.713
Overall	0.650

## Discussion

The main finding of this study was that Bigliani’s classification lacks intra- and inter-observer reliability for assessment of acromial morphology despite a sophisticated imaging modality like MRI. Association between rotator cuff disorders and acromial morphology is evident; however, Bigliani’s classification lacks sufficient accuracy for clinical guidance. Therefore, shoulder surgeons should consider using different radiological parameters to evaluate shape of acromion.

Neer’s theory of extrinsic impingement leading to cuff disorder was followed by the opinion of clinical significance of acromial morphology which was first described by Bigliani e*t al*^6^. Many studies confirmed the association of excessive acromial coverage and rotator cuff disorders^16-19^. However, debate still exists about the pathogenesis of SIS and rotator cuff disorders between mechanical attrition of the tendon and primary cuff tendinopathy. Currently, it is known that hook shaped acromion is an acquired transformation of acromial morphology rather than a congenital anatomical variation which is highly associated to age^20^. In their cadaveric study, Bigliani *et al* classified acromion morphology into three types and reported an association between acromion shape and rotator cuff disease. However, advanced mean population age of this study also supports this theory and could explain high reported rates of type II and III acromions. There are also other studies suggesting that anatomic changes of acromion are the results of degenerative rotator cuff process rather than the cause^21-23^. However, acromial morphology still plays a very important role in clinical evaluation of patients with rotator cuff pathology and classification described by Bigliani *et al* is still most commonly used technique to assess acromial anatomy in clinical practice.

In previous studies, it has been shown that acromial insertion of coracoacromial ligament is the site for enchondral ossification and spur formation process in cases with degenerative rotator cuff disease^22,24^. Since coracoacromial ligament acts as a buffer against superior translation of humeral head^25^, it is thought that an incompetent rotator cuff could cause tension on the ligament and lead to traction spur formation^25^, consequently contribute to impingement. However, the spur is not a portion of native acromion and does not significantly alter dimensions or morphology of the acromion^23^. But the presence of a spur may cause difficulty to classify the acromion as the true acromial edge may appear indistinct on outlet view radiographs. Another problem in classifying the acromial shape is the technical difficulty to obtain a reproducible outlet view. Morrison and Bigliani reported good correlation between acromial morphology and rotator cuff tears on outlet view radiographs obtained by a single technologist^26^. In their subsequent study, they showed a significant decrease in correlation when the radiographs are taken by different technologists^27^. Other authors also reported that changes of the radiographic projection may convert type II acromion appearance into type I and cause classification problems^28^.

Another disadvantage of Bigliani’s classification is lack of standardised objective assessment criteria and is that this system depends on naked eye observation. In order to have a more reliable and reproducible classification, Park *et al* defined standardised objective criteria for evaluation of acromial shape on outlet radiographs^10^. They reported higher inter-observer agreement rating compared to Bigliani’s criteria but intra-observer reliability coefficient of both groups were comparable. Authors concluded that a more reliable classification is possible with more definitive and objective evaluation, especially for distinction of type II and III acromion.

Reliability of Bigliani’s classification has been assessed on several previous studies in literature. Consistently with our results, Bright *et al* reported a fair overall inter-observer reliability (κ=0.35) and a moderate intra-observer repeatability (κ=0.55). Intra-observer repeatability was not significantly different between different levels of expertise^11^. In another study, Jacobson *et al* reported higher inter- and intra-observer reliability coefficients (κ=0.52 and 0.88, respectively)^9^. In this study, all observers were experienced fellowship-trained shoulder surgeons which may explain higher inter- and intra-observer reliability coefficient values. Results of mentioned study also showed that type I acromion was easily classified but delineation between type II and type III may be problematic, and authors concluded that more objective and standardised criteria are needed.

When MRI was introduced into clinical practice, it was proposed as an alternative imaging modality to assess acromial morphology. Since it is a tomographic method, it was expected to be superior to conventional outlet radiographs and provide more accurate results due to projection errors and technique variations related to radiographs. However, it has been shown that radiographs had higher correlation and inter-observer reliability compared to MRI^13^. These results were attributed to the fact that selection of slice position would affect acromial shape seen on MRI. Mayerhoefer et al assessed reliability of MRI to determine acromial morphology with different slice positions^12^. Results of this study showed that highest reliability was obtained with MRI slice positioned just lateral to the acromioclavicular joint when a single MRI slice was used. However, conventional radiography was superior to all single MRI slice positions unless multiple slice positions were used. In our study, single slice position recommended by Mayerhoefer *et al*^12^ was used to assess acromial shape. Accordingly, to their findings, assessment of acromial morphology on MRI did not show superior reliability. Moreover, inter- and intra-observer agreement did not differ according to level of expertise of observers.

## Conclusion

Findings of the present suggest that despite a sophisticated imaging modality like MRI, Bigliani’s classification apparently lacks accuracy and additional criteria, or different assessment methods are required to assess acromial morphology for clinical guidance. As a matter of fact, different radiological assessment methods to evaluate multiplanar acromial morphology have been described in recent studies^29,30^. We think that shoulder surgeons should not rely only commonly used Bigliani’s classification and make use of combination of these other methods as well to understand the association between shoulder anatomy and rotator cuff disorders and for clinical decision making.
